# Multimodal Approach to Assessment of Fecal Microbiota Donors based on Three Complementary Methods

**DOI:** 10.3390/jcm9072036

**Published:** 2020-06-29

**Authors:** Jaroslaw Bilinski, Mikolaj Dziurzynski, Pawel Grzesiowski, Edyta Podsiadly, Anna Stelmaszczyk-Emmel, Tomasz Dzieciatkowski, Lukasz Dziewit, Grzegorz W. Basak

**Affiliations:** 1 Department of Hematology, Oncology and Internal Medicine, Medical University of Warsaw, 02-091 Warsaw, Poland; grzegorz.basak@wum.edu.pl; 2 Department of Environmental Microbiology and Biotechnology, Institute of Microbiology, Faculty of Biology, University of Warsaw, 02-096 Warsaw, Poland; 3 Foundation for the Infection Prevention Institute, 02-991 Warsaw, Poland; paolo@fipz.edu.pl; 4 Department of Microbiology, Institute of Medical Sciences, University of Rzeszów, 35-310 Rzeszów, Poland; edyta.podsiadly@uckwum.pl; 5 Department of Laboratory Diagnostics and Clinical Immunology of Developmental Age, Medical University of Warsaw, 02-091 Warsaw, Poland; anna.stelmaszczyk-emmel@wum.edu.pl; 6 Department of Medical Microbiology, Medical University of Warsaw, 02-091 Warsaw, Poland; dzieciatkowski@wp.pl

**Keywords:** fecal microbiota transplantation, feces donor, fecal microbiota, flow cytometry, viability of bacteria, next-generation sequencing, culturing of fecal microbiota

## Abstract

Methods of stool assessment are mostly focused on next-generation sequencing (NGS) or classical culturing, but only rarely both. We conducted a series of experiments using a multi-method approach to trace the stability of gut microbiota in various donors over time, to find the best method for the proper selection of fecal donors and to find “super-donor” indicators. Ten consecutive stools donated by each of three donors were used for the experiments (30 stools in total). The experiments assessed bacterial viability measured by flow cytometry, stool culturing on different media and in various conditions, and NGS (90 samples in total). There were no statistically significant differences between live and dead cell numbers; however, we found a group of cells classified as not-dead-not-alive, which may be possibly important in selection of “good” donors. Donor C, being a regular stool donor, was characterized by the largest number of cultivable species (64). Cultivable core microbiota (shared by all donors) was composed of only 16 species. ANCOM analysis of NGS data highlighted particular genera to be more abundant in one donor vs. the others. There was a correlation between the not-dead-not-alive group found in flow cytometry and *Anaeroplasma* found by NGS, and we could distinguish a regular stool donor from the others. In this work, we showed that combining various methods of microbiota assessment gives more information than each method separately.

## 1. Introduction

Stool suspension is commonly used as a straightforward, cheap and non-invasive material for treating several conditions and diseases. Outcomes of fecal microbiota transplantation (FMT) are encouraging in many diseases (e.g., inflammatory bowel diseases [1]) and excellent in others (e.g., *Clostridioides difficile* infection, especially as a life-saving therapy [2,3]). Not surprisingly, there are more than 250 FMT clinical trials completed or ongoing worldwide [4].

Despite the thriving of the FMT trials, our knowledge on the real composition of “healthy microbiota” is still scarce. We do not exactly know how the transplanted bacteria survive, colonize, and function in the recipient’s gut or, most importantly, which methods may, or should, be used to diagnose and monitor transplanted feces to assess whether the fecal microbiota solution is appropriate for transplantation and consists of a “healthy microbiota”. We also acknowledge that a healthy gut microbiota requires proper virus and fungi composition [5,6].

Analyses of human intestinal microorganisms were, until recently, mostly performed by culture-dependent methodologies, limiting the screened biodiversity only to the cultivable species, although it is known that only about 15–20% of microbes living in the human gut are cultivable as of now. The availability of novel tools, primarily next-generation sequencing (NGS), has enabled the assessment of marker taxonomical genes and even whole genomes retrieved from the complex microbial communities. The most widely used NGS method for the taxonomic and phylogenetic evaluation of bacterial community composition relies on 16S rRNA gene PCR amplicon analysis [7,8,9].

Currently, it can be observed that the market offering fecal microbiota suspensions and tools for FMT suspensions assessments is growing rapidly. Companies provide material from different donors, using various methodologies for testing the donors and stool processing. However, each of these methods is usually applied separately, and on these distinct analyses far-reaching conclusions are built. There are very few reports comparing different methods for the assessment of gut microbiota [10,11,12,13] and indicating how to assess the stability of fecal microbiota in the donors. There is also a need to identify “super-donor” units, i.e., persons whose microbiota contains all relevant microbes and potentially can cure the vast majority of microbiota-related diseases and conditions [14].

There is also a need for defining “super-donors” for FMT. It is postulated that there are individuals whose gut microbiome possesses certain characteristics, such as the presence or absence of specific (unfortunately not fully recognized) bacteria, phages and metabolites, which protect the donor from the vast majority of gut-related dysbiosis [14]. Furthermore, it is postulated that these individuals are the most desired donors (namely “super-donors”), and that their fecal microbiota is highly suitable for transplantation. Theoretically, such donors should be adequate for every FMT intervention. However, recent studies prove that there are some (still unknown) specific features making these (super) donors not so universal [15,16]. Therefore, an algorithm for finding perfectly matching donor–recipient couples still needs elucidation. We believe that this coupling of donor and recipient may be possible, but this requires more data and more diagnostic/analytical tools. To define what exactly “super-donor” means, we need to have more well-designed comparable clinical trials for dysbiosis-related diseases [17]. Only then will we be able to conduct a general investigation, looking for features common to all donors. Our study is in line with this general quest in medicine. Summarizing, for the purpose of this study, the term “super-donor” was used with its complex definition, including an indication that the microbiota of the “super-donor” has all the necessary beneficial components to maintain human welfare and that it will most probably cure the majority of dysbiosis. Besides, the most important conclusion must be the statement that the donor can be defined as a “super-donor” only when the majority (ideally all) clinical outcomes of FMT (from this donor) are good.

In this study, we conducted a series of experiments using a multi-method approach to trace the stability of the composition of the gut microbiota in various donors over time and to find the most suitable method for assessing the quality of the gut microbiota for the proper selection of fecal donors and to pave the way to find “super-donors”. Moreover, we were looking for bacterial indicators of “good” and/or “bad” donors to simplify and parametrize the selection of suitable givers of stool samples for FMTs. We hypothesized that various methodologies of microbiota assessment to evaluate donors gives more data than each method separately. 

## 2. Material and Methods

### 2.1. Stool Donors 

Ten consecutive stools donated by each of three donors were used for the experiments (30 stools in total). The donors of stool were randomly selected males (donors named A and B) and an intentionally chosen male (donor C, that is, a regular stool donor registered in the Polish stool bank). They were selected according to our protocol published previously [18]. Donor C was selected with respect to criteria described by Cammarota et al. [19]. All donors were screened by a questionnaire based on international guidelines that was presented in our previous publication [18]. Briefly, one of them (donor C; male, 28 years old, healthy, with normal BMI) was a regular donor of feces, for the purpose of producing a preparation for fecal microbiota transplantation (chosen from the stool donor bank) and the other two were randomly selected males (donor A—male, 16 years old with food allergy, recurrent aphthous stomatitis and normal BMI, and donor B—male, 55 years old, a medical worker with inhaled allergy and a BMI of 27). A medical questionnaire with basic data was received from each person. Each feces sample was prepared in the same time frame and in the same way by homogenizing, diluting in normal saline, and sieving through sterile gauze or sieves to obtain a clear, homogeneous fluid being a suspension of feces. This is the regular way of producing feces for use as FMT [18]). The material prepared in this way was divided into three parts—one for assessment by flow cytometry in the LIVE/DEAD method (Molecular Probes, Eugene, OR, USA), the other for performing classical culturing, and the third for immediate isolation of DNA for V3V4 16S rDNA variable region sequencing (90 samples in total).

### 2.2. Flow Cytometry

**Figure 1:**
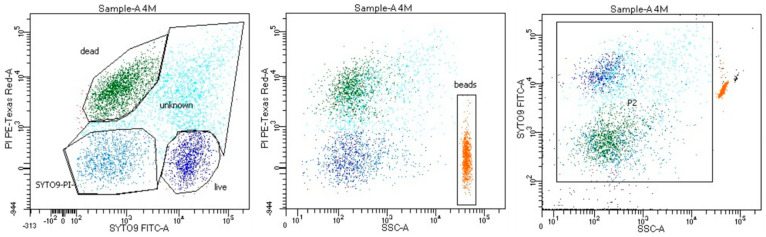
Gating strategy shown on one of the samples. SYTO9-positive PI-negative cells were considered alive, SYTO9-negative PI-positive cells were considered dead, other cells were considered as unknown, with special gating on SYTO9-negative PI-negative cells, which were called “double negative” cells.

Bacterial viability in samples was measured by flow cytometry using the LIVE/DEAD BacLight™ Bacterial Viability and Counting Kit (L34856, Molecular Probes) according to manufacturer instructions (Molecular Probes) [20]. Briefly, 977 µL of 0.9% NaCl, 1.5 µL of SYTO9, 1.5 µL of propidium iodide (PI) and 10 µL of diluted sample were added to a flow cytometry analysis tube. Samples were 10-fold diluted in 0.9% NaCl. The tube was incubated for 15 min in a dark at room temperature. A quantity of 10 µL of the microsphere suspension (beads) was added to the stained sample. The total volume of the sample in the flow cytometry analysis tube was 1000 µL. The samples were analyzed on a LSR Fortessa flow cytometer (Becton Dickinson, Franklin Lakes, NJ, USA) with FACS Diva v8 software (Becton Dickinson). The gating strategy is shown in Figure 1 and shows three main cell populations—alive, dead and unknown (probably alive, probably dead) with a special “double negative” group of cells (SYTO9^−^PI^−^). The number of bacteria per mL in each analyzed gate was counted according to the following formula taken from the manufacturer materials:

$$\frac{\left(\left(\#\;ofevents\in gatedbacteriaregion\right)\times \left(dillutionfactors\right)\right)}{\left[\left(\#\;ofevents\in beadregion\right)\times 10^{-6}\right]}=bacteria/\mathrm{mL}$$

### 2.3. Cultivation of Stool Microbiota

Samples were plated on six different agar media and incubated under conditions as follows. (i) CNA medium (colistin nalidixic acid agar; Oxoid, Basingstoke, UK) for cultivation of Gram-positive aerobes, an enriched agar medium containing sheep's blood, colistin and nalidixic acid (to inhibit the growth of Gram-negative bacteria). Culture conditions: aerobic with 5% CO_2_, 37 °C, 48 h. (ii) MacConkey medium (bioMérieux, Marcy l’Etoile, France) for the isolation of Gram-negative rods, containing bile salts and crystal violet (to inhibit the growth of Gram-positive bacteria). Culture conditions: aerobic, 37 °C, 48 h. (iii) Bile and esculin (CC) medium (Oxoid)—a medium intended for the isolation and identification of bacteria belonging to the genus *Enterococcus*, which grow well in the presence of bile and have the ability to break down esculin. Culture conditions: aerobic, 37 °C, 48 h. (iv) Schaedler Anaerobe KV Selective Agar with freeze-dried horse blood and the addition of kanamycin and vancomycin (bioMérieux)—a highly nutritious medium for the selective growth and isolation of anaerobic bacteria, especially of the genus *Bacteroides* and *Prevotella*. Culture conditions: anaerobic, 37 °C, 4 days. (v) Schaedler Anaerobe KV Selective Agar with freeze-dried horse blood (bioMérieux)—a highly nutritious medium for the isolation of absolute and relative anaerobes. Culture conditions: anaerobic, 37 °C, 4 days. (vi) Sabouraud agar with gentamicin and chloramphenicol (Oxoid)—selective medium for cultivation of mold and yeast, high glucose concentration. The presence of antibiotics (chloramphenicol and gentamicin) and acidic pH inhibits bacterial growth; the presence of antibiotics is another selection factor. Culture conditions: aerobic, 37 °C, 10 days. The anaerobic incubations were carried out in anaerobic jars and atmosphere generators (Oxoid).

After the initial sample processing, colonies were selected (at least one colony per morphology) for identification using a Microflex LT mass and MBT Compass IVD Biotyper software (Bruker Daltonics, Bremen, Germany). The colonies were deposited on a MALDI-TOF (Bruker Daltonics) target microflex and extracted with 5% formic acid, air dried and then overlaid with 1 µL matrix solution of α-cyano-4-hydroxycinnamic acid in 50% acetonitrile and 2.5% trifluoroacetic acid. Two spots were examined for each colony. The Biotyper software was used to compare the protein profile of the cultured bacteria from a database of Bruker consisting of 2750 of protein profiles. A score > 1.9 was considered a high-level identification of a species, a score >1.7 indicated the identification of a genus. Strains of bacteria with scores lower than 1.7 were considered unidentified. 

To enumerate the number of colony-forming units (CFU) in the stool samples, 0.2 g of stool was diluted in 1 mL of phosphate-buffered saline (PBS), and 1–5 µL of watery sample was spread on each media. Bacterial counts were recorded as CFU per gram of feces for each isolated species.

### 2.4. DNA Sequencing

Total bacterial DNA was extracted using a Qiagen DNeasy Power Soil kit (Qiagen, Hilden, Germany) according to the manufacturer’s instructions and stored at −20 °C. Using isolated DNA as a matrix, PCR reactions were performed in triplicate (to reduce PCR bias) using a Bakt_341F 5′-CCTACGGGNGGCWGCAG-3′ and Bakt_805R 5′-GACTACHVGGGTATCTAATCC-3′ primer pair amplifying the variable V3 and V4 regions of the 16S rRNA genes [21,22]. Electrophoretic analysis was performed for each of three replicates for qualitative and quantitative evaluation of the PCR products. Then, products of three independent PCR reactions for each sample were mixed and used for the DNA sequencing as one amplicon to minimize the error due to the selectivity of the PCR reactions. The amplified PCR products were sequenced using an Illumina MiSeq instrument (Illumina, San Diego, CA, USA) in paired-end mode using a v3 chemistry kit (Illumina) at BIOBANK LAB (Chair and Department of Molecular Biophysics, University of Lodz, Łódź, Poland).

### 2.5. Bioinformatic Analysis

Sequencing data were subjected to NonPareil 3 [23] for sequencing depth assessment and later processed with Qiime2 (version 2018.11) package [24]. The reads were imported into Qiime2 and run through the dada2 plugin to obtain amplicon sequence variants (ASV) [25]. Taxonomy was assigned for each of the ASVs using a pre-trained naive Bayes classifier, based on the Silva 132 99% database [26], which was trimmed to include only the V3 and V4 regions of the 16S rRNA gene, bound by the Bakt_341F and Bakt_805R primer sequences. Alfa and beta diversity metrics were generated using the following Qiime2 plugins: phylogeny (including mafft aligner and FastTree tool), diversity and emperor [27,28,29].

### 2.6. Statistical Analysis

In order to identify genera that differ in abundance between samples from different donors, ANCOM analysis was used [30]. All additional statistics were generated using R (version 3.5.1) in the RStudio environment. Libraries such as ggplot2, cowplot and ggpubr were used for data visualization [31,32,33]. Except when otherwise stated, p-values of less than 0.05 were considered statistically significant.

### 2.7. Sequencing Data Availability

Next-generation sequencing data has been deposited and is available under the number PRJEB36368 and link www.ebi.ac.uk/ena/data/view/PRJEB36368.

### 2.8. Ethics

All subjects gave their informed consent for inclusion before they participated in the study. The investigations were carried out following the rules of the Declaration of Helsinki. According to local bioethics committee rules for non-intervention studies, no approval was needed to conduct this study.

## 3. Results

### 3.1. Analyses Applying the Flow Cytometry

**Figure 2:**
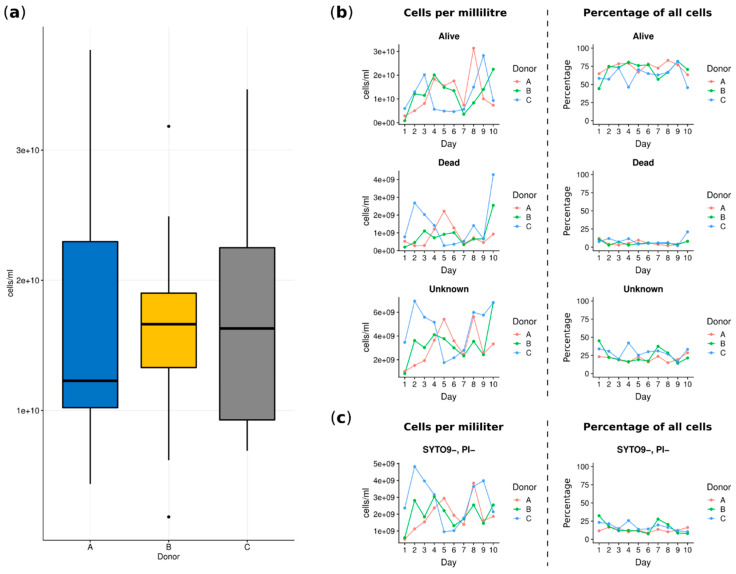
Cytometry cell count charts. (**a**) Total cell count per donor summed over all samples. (**b**) Charts depicting variability in collected samples per day per donor. The first column shows absolute counts of cells classified as alive, dead or unknown. The second column shows cell counts as a percentage of the total number of cells counted in a given sample. (**c**) Two charts showing the variability of cells classified as a subgroup of unknown clusters: SYTO9-, PI-. The percentage was calculated versus the unknown group cell count.

The flow cytometry analysis allowed evaluation of the total number of cells in each sample as well as live versus dead cells. This analysis was used to answer the question whether the number of bacterial cells is important in the evaluation of fecal microbiota and can be used as an estimator of a “good” or “bad” donor. The performed analysis showed that there were no statistically significant differences between cell numbers in the fecal microbiota suspensions prepared from the donors’ stools (see Figure 2a). The summarized average cell count of all samples was equal to 1.664 × 10^10^ cells/mL, (±0.913 × 10^10^ cells/mL). Looking at numbers of live, dead and unknown cells each day for each donor, we noted discrete differences in the number of cells per mL of stool suspension; however, this was not statistically significant (donor A: mean 1.627 × 10^10^, ±1.015 × 10^10^; donor B: mean 1.632 × 10^10^, ±0.852 × 10^10^; donor C: mean 1.732 × 10^10^, ±0.96 ×10^10^). As shown in Figure 2b (right column), the percentages of all fractions of cells, i.e., alive, dead, unknown (not stained with one of the reagents) and SYTO9-PI- (subgroup of “unknown”, not stained by both reagents considered as “double negative”) showed relatively stable numbers in each stool sample per day and per donor. The noticeable domination of the alive cells was observed (Figure 2b). 

In the next step, we specifically focused on alive cells, searching for if the number of this group of bacterial cells is important for the evaluation of fecal microbiota. As we observed, there were no significant differences in the viability of cells for each donor, and alive cells accounted for similar average percentages (Table S1).

### 3.2. Cultivation of Stool Microbiota—Classical Microbiological Evaluation 

**Figure 3:**
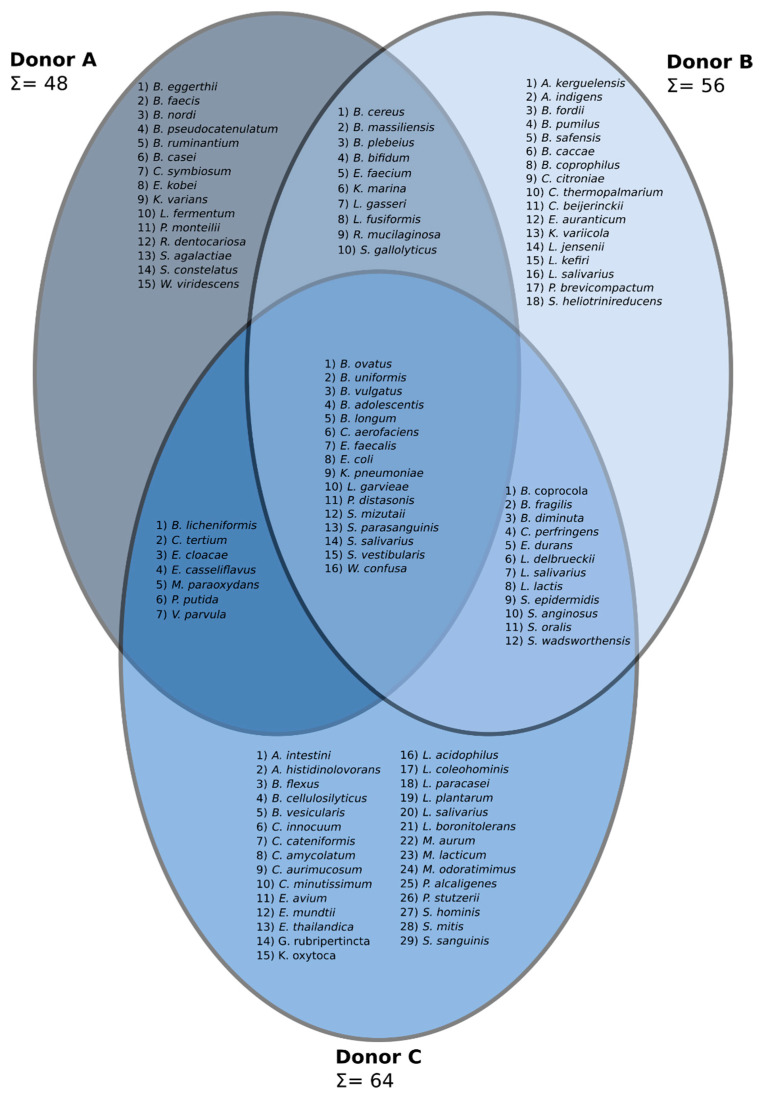
A three-set Venn diagram constructed based on the data from a classical microbiology approach. Identified genera are as follows: *Acidaminococcus* (*A. intestini*), *Arthrobacter* (*A. histidinolovorans*, *A. kerguelensis*), *Azoarcus* (*A. indigens*), *Bacillus* (*B. cereus*, *B. flexus*, *B. fordii*, *B. licheniformis*, *B. pumilus*, *B. safensis*), *Bacteroides* (*B. caccae*, *B. cellulosilyticus*, *B. coprocola*, *B. coprophilus*, *B. eggerthii*, *B. faecis*, *B. fragilis*, *B. massiliensis*, *B. nordi*, *B. ovatus*, *B. plebeius*, *B. uniformis*, *B. vulgatus*), *Bifidobacterium* (*B. adolescentis*, *B. bifidum*, *B. longum*, *B. pseudocatenulatum*, *B. ruminantium*), *Brevibacterium* (*B. casei*), *Brevundimonas* (*B. diminuta*, *B. vesicularis*), *Clostridium* (*C. beijerinckii*, *C. citroniae*, *C. innocuum*, *C. perfringens*, *C. symbiosum*, *C. tertium*, *C. thermopalmarium*), *Collinsella* (*C. aerofaciens*), *Coprobacillus* (*C. cateniformis*), *Corynebacterium* (*C. amycolatum*, *C. aurimucosum*, *C. minutissimum*), *Enterobacter* (*E. cloacae*, *E. kobei*), *Enterococcus* (*E. avium*, *E. casseliflavus*, *E. durans*, *E. faecalis*, *E. faecium*, *E. mundtii*, *E. thailandica*), *Escherichia* (*E. coli*), *Exiguobacterium* (*E. auranticum*), *Gordonia* (*G. rubripertincta*), *Klebsiella* (*K. oxytoca*, *K. pneumoniae*, *K. variicola*), *Kocuria* (*K. marina*, *K. varians*), *Lactobacillus* (*L. acidophilus*, *L. coleohominis*, *L. delbrueckii*, *L. fermentum*, *L. gasseri*, *L. jensenii*, *L. kefiri*, *L. paracasei*, *L. plantarum*, *L. salivarius*), *Lactococcus* (*L. garvieae*, *L. lactis*), *Lysinibacillus* (*L. boronitolerans*, *L. fusiformis*), *Microbacterium* (*M. aurum*, *M. lacticum*, *M. paraoxydans*), *Myroides* (*M. odoratimimus*), *Parabacteroides* (*P. distasonis*), *Penicillium* (*P. brevicompactum*), *Pseudomonas* (*P. alcaligenes*, *P. monteilii*, *P. putida*, *P. stutzerii*), *Rothia* (*R. dentocariosa*, *R. mucilaginosa*), *Slackia* (*S. heliotrinireducens*), *Sphingobacterium* (*S. mizutaii*), *Staphylococcus* (*S. epidermidis*, *S. hominis*), *Streptococcus* (*S. agalactiae*, *S. anginosus*, *S. constelatus*, *S. gallolyticus*, *S. mitis*, *S. oralis*, *S. parasanguinis*, *S. salivarius*, *S. vestibularis*), *Stretococcus* (*S. sanguinis*), *Sutterella* (*S. wadsworthensis*), *Veillonella* (*V. parvula*), *Weissella* (*W. confusa*, *W. viridescens*).

A complex cultivation experiment was performed to evaluate whether this technique can reveal culturable bacterial indicators for “good” versus “bad” stool donors. In total, 104 species representing 36 genera were found. The summarized bacterial species composition for each donor is indicated (Figure 3). Presentation of data in the form of a Venn diagram enabled us to indicate bacterial species that were characteristic of each donor, species that were shared by two donors and, finally, species that created a core microbiota and were found in all analyzed donors (Figure 3). Clearly, we can see that donor C, being a regular stool donor, is characterized by the largest number of cultivable species (64) obtained from his stool. Samples from other donors had lower numbers of cultivable species (48 and 56, respectively). Moreover, in the stool samples collected from donor C, the largest number of unique species (29) was found. Interestingly, the cultivable core microbiota was composed of only 16 species. 

**Figure 4:**
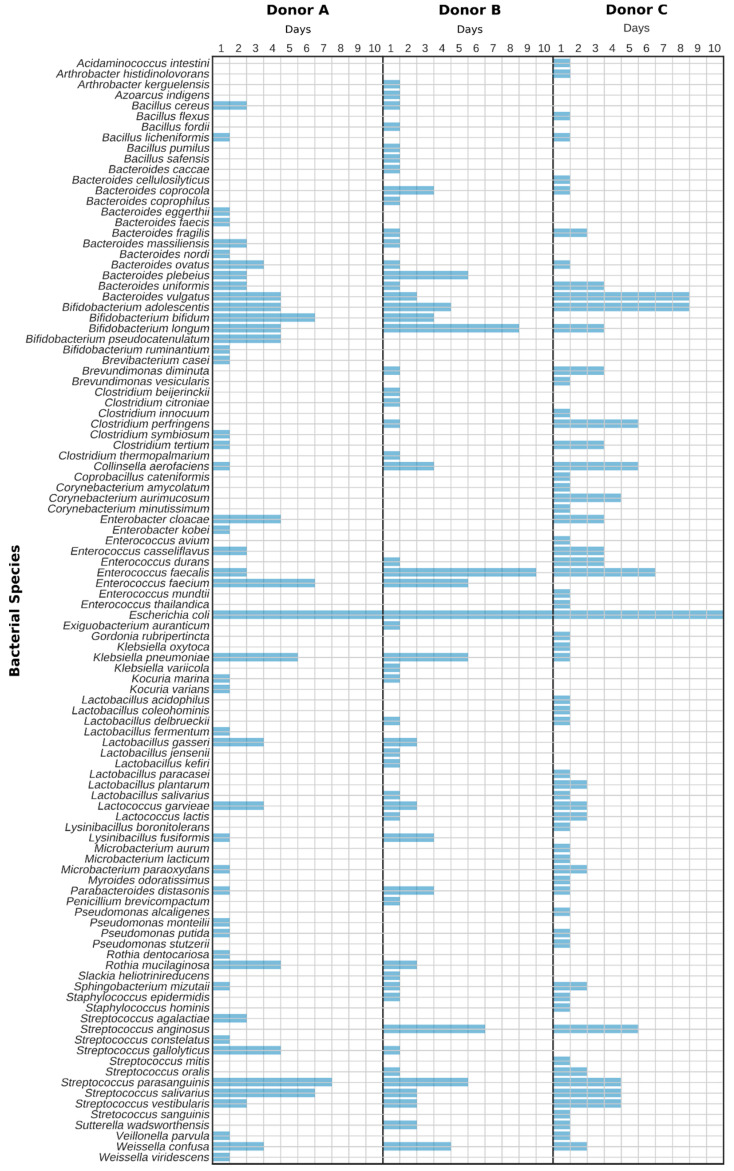
Diagram showing the daily presence of the particular cultivable bacterial species in stool samples.

In the next step, we evaluated the presence of identified species over time, i.e., throughout 10 sampling days (Figure 4). It was shown that the most persistent species was *Escherichia coli*, being detected in all samples. Other species, such as *Enterococcus faecalis*, *Streprococcus parasanguinis*, *Bifidobacterium adolescentis*, *Enterococcus faecium* and *Streptococcus salivarius* were also detected in the majority of samples, yet their persistence varied greatly between donors (Figure 4). It is noticeable that a plethora of bacterial species occurred only on individual days. This may be a consequence of the bias of this method or of a simple one-time variation depending on the food consumed. Therefore, when using conventional culturing as an evaluation strategy for the assessment of the quality of stool samples, it is important to repeat sampling from particular donors for several days. Single sampling can deliver non-representative and possibly false results. 

### 3.3. Next-Generation Sequencing

A total of 5,694,140 reads were obtained from Illumina MiSeq sequencing, with reads per sample ranging from 111,656 to 291,029. Quality control and merging of paired-end reads using the dada2 software package resulted in the retention of, on average, 47.74% (σ = 2.21) reads per sample (Table S2). Both the Nonpareil 3 and alpha rarefaction analysis (Qiime2 diversity plugin) showed sequencing depths close to 100%.

**Figure 5:**
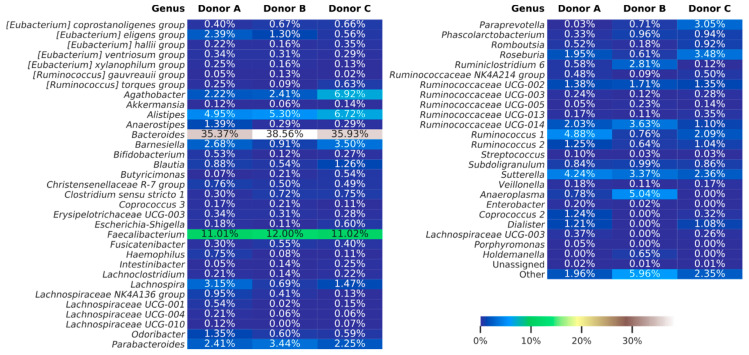
Heat map showing bacterial genera detected using amplicon sequencing (V3–V4 region of 16S rDNA). The summarized data for each donor are presented. The “others” group summarizes genera with individual abundances lower than 0.5% in any sample. Sequences unassigned at the genera taxonomy level were grouped and named “unassigned”.

Merged reads subjected to further analyses were dereplicated into 9868 amplicon sequence variants (ASV), with the number of reads for ASV ranging from 1 to 10,098. Taxonomy assignment based on the Silva database (release 132), showed 97.75% of ASVs classified down to the genus level. Overall classification showed that 99.98% of all reads were bacterial, 0.01% archeal and less than 0.01% were unclassified. The bacterial ASVs represent 18 classes, with Bacteroidia and Clostridia in relative abundance, constituting averages of 49.9% and 40.0%, respectively. At a genus level, the most dominant taxa were *Bacteroides* and *Faecalibacterium*, with relative abundance in each sample no less than 35% and 11%, respectively. The data was not analyzed on species level because a single marker region does not allow this kind of resolution [34]. Figure 5 shows the abundances of each taxon identified in stool samples. 

**Figure 6:**
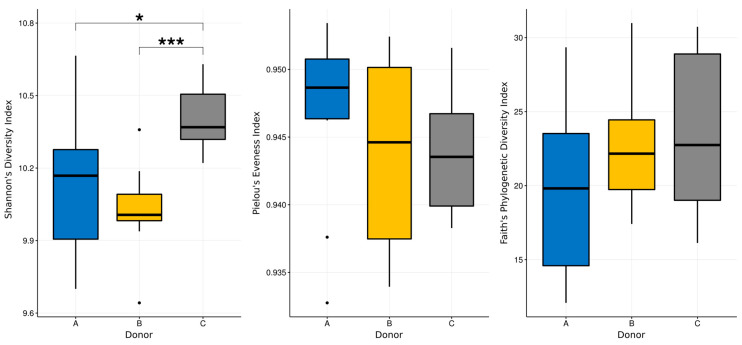
Boxplots showing selected biodiversity indices calculated for the data from the metabarcoding analysis. Kruskal-Wallis test was used to detect statistically significant differences. *—*p*-value less than 0.01; ***—*p*-value less than 0.001.

The Shannon diversity index along with Pielou’s evenness index have been calculated for all of the samples, and the Kruskal–Wallis test was used to determine if there were any statistical differences between them. The Pielous evenness index for all of the samples was relatively high, ranging from 0.94 to 0.95, and no statistical differences between donors were detected. Interestingly, the Shannon index was similar for donors A and B, with its mean values equal to 10.11 and 10.02, while it was slightly, but significantly, higher for donor C—10.39 (*p* = 0.0191 for donor A versus C and *p* = 0.0005 for donor B versus C according to Kruskal–Wallis test, H value = 12.18; see Figure 6).

Given that the above statistical test for significant differences yielded two pairs, donor A versus C and donor B versus C, these pairs were subjected to ANCOM analysis. In the first case, ANCOM analysis highlighted two Gram-negative, obligatory anaerobe genera to be more abundant in samples from donor C; these were *Acidaminococcus* and *Paraprevotella*. The same analysis showed *Anaeroplasmatales* and *Gastranaerophilales* orders to be more abundant in samples from donor A.

An ANCOM analysis of the second pair (donor B versus C) highlighted *Anaeroplasma* as more abundant in donor B, along with *Holdermanella* genera and two, not well described, bacterial families—*Muribaculaceae* and *Puniceicoccaceae*, members of the *Bacteroidetes* and *Verrucomicrobia* phyla, respectively. As for taxa more abundant in the donor C microbiota, two members of the *Firmucutes* phylum were detected: *Lachnospiraceae* and *Dialister*.

**Figure 7:**
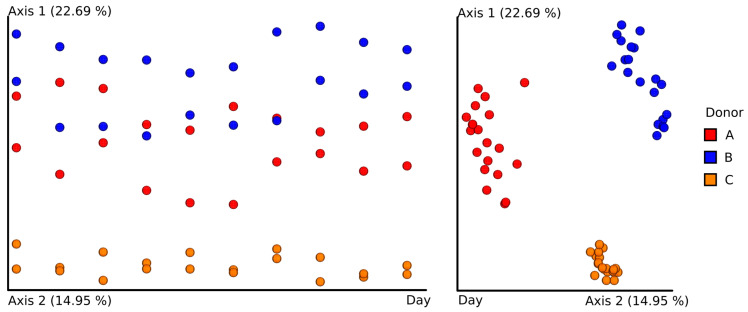
Principal coordinates analysis (PCoA) visualization. The PCoA was built using the Bray–Curtis dissimilarity index with day of sample collection as one axis.

As was done for classical culturing experiments, the stability of the intestinal microbiota over time was assessed. Although no statistically significant differences were detected, principal coordinates analysis (PCoA) on the Bray–Curtis dissimilarity index shows that the overall internal similarity of time-resolved samples from donor C was much higher than for other donors. Clustering the bacterial composition of feces in donor C indicates the most stable composition of intestinal microbiota over time (Figure 7).

Pearson correlation coefficients between the double negative group of cells (SYTO9-, PI-) and genera-level taxonomy data showed that relative abundance of *Anaeroplasma* is positively correlated with the double negative group per sample percentage (ρ = 0.6312), followed by *Sanguibacteroides* (ρ = 0.4592). 

## 4. Discussion

In this study, we used a multi-method approach to (i) test a hypothesis that each method separately may be useful in donor characterization and to (ii) try to distinguish a regular donor of feces from the stool bank (as we know his stool is beneficial for patients and he is generally healthy and meets all inclusion criteria for being a stool donor) from randomly chosen persons. 

Although very complex, our analysis should be still considered as a preliminary one with several limitations. First of all, we tested only three donors in total in our study; however, our goal was to perform complex analysis with screening of multiple samples per patient in a time-resolved manner. Additionally, a relatively weak point of our analysis could also be using a limited number of media for the cultivation experiment; however, we carefully chose them to cover both aerobic and anaerobic taxa.

Taking into account all limitations, our work showed that assessment of the intestinal microbiota is very complex. Each of the applied methods separately had significant limitations, however, enabled some significant observations. Classic bacterial culturing showed that single intestinal microbiota sampling can give false, and surely not representative, results, because many bacteria are found in the feces irregularly, and the composition of fecal microbiota may, to some extent, change daily [35]. For a complete picture of the composition of the intestinal microbiota, multiple culturing should be performed, and obtained results should be analyzed together. The next applied procedure, i.e., flow cytometry, showed that live bacteria dominated the samples. However, discussion on the active component of the intestinal microbiota preparation continues. It is said that live bacteria are not necessarily responsible for the beneficial properties of intestinal microbiota, and metabolites or even individual genes should be taken into consideration [36]. Nevertheless, the prevalence of living bacteria is a positive aspect in the argument that live bacteria are the factor that restores intestinal homeostasis [37]. The last of the applied methods, i.e., next-generation sequencing, definitely brings the most abundant data, considering the bacterial diversity of the intestinal microbiota. The weakness of this method, however, is that the results are relative; we do not have absolute numbers of individual genera and the bacteria represented in a very low titer may be underrepresented in the sample [38]. Keeping in mind that this is a semi-quantitative method, not a quantitative one, and inferences based on the indicated percentages must be careful, we can see the differences that we observed between donor C and donors A and B may point to unique combinations of individual species. Possibly, the key species for health processes are present in donor C, who is a regular donor of feces. Such conclusions will always be arbitrary, but the “beneficial” species described in the literature, such as *Blautia, Barnesiella, Butiricimonas* [18] and *Roseburia*, are significantly more abundant in donor C than in others; however, other strains considered beneficial, such as *Bacteroides* and *Faecalibacterium*, occur in comparable percentages (Figure 5).

More definite conclusions can be drawn when looking collectively at the results obtained with the application of all these methods. In general, the metabarcoding analysis revealed that Shannon’s biodiversity index was significantly higher for donor C than others. This is in agreement with previous results, indicating that biodiversity is one of the most important markers of a healthy microbiome [39,40]. There are no specific values of diversity indexes that particular samples of intestinal microbiota should achieve in order to be recognized as more valuable than others. However, high biodiversity indicates a richer intestinal flora and is associated, according to current knowledge, with health [14].

Regarding a microbiota composition assessed by classical culturing and NGS, we found some species and genera being unique for donor C, and it was also found that abundances of particular species may play a role. The donor C microbiota was most stable in time, which may also be an indicator of a super-donor. However, even in donor C’s stool samples, we observed some day-to-day diversity. A possible lack of influence of this variability on overall microbiotal homeostasis may be explained by the hypothesis that a healthy “functional core” of the microbiome is maintained by a complement of metabolic and other molecular functions that are performed by the microbiome within a particular habitat but are not necessarily provided by the same organisms in different individuals [41]. Therefore, according this hypothesis, the microbiome as a pool of genes remains stable.

In this study, we identified genera (*Roseburia, Barnesiella, Blautia, Butiricimonas*) considered beneficial as more abundant in donor C than in the others. ANCOM analysis on samples from donor A versus C highlighted *Acidaminococcus* and *Paraprevotella* to be more abundant in samples from donor C. Both of these genera are common human gut microbiota, with the former shown to be able to use glutamic acid as the only source of energy and produce hydrogen and hydrogen sulfide from citrate [42,43]. *Paraprevotella*, on the other hand, is an opportunistic human pathogen, associated with non-alcoholic fatty liver disease [44,45]. The same analysis has shown *Anaeroplasmatales* and *Gastranaerophilales* orders to be more abundant in samples from donor A than from donor C. While not much is known about the *Gastranaerophilales* order, *Anaeroplasmatales* is a Gram-negative obligatory anaerobe, a member of Mollicutes class, which also harbors some human pathogens such as *Mycoplasma* and *Ureaplasma*.

The same analysis of the second pair (donor B versus C) also highlighted *Anaeroplasma* in this setting to be more abundant in donor B, along with the *Holdermanella* genera, a Gram-positive member of the Firmicutes phylum associated with chronic kidney disease [46]. As for taxa more abundant in the donor C microbiota, two members of the Firmucutes phylum were detected: *Lachnospiraceae* and *Dialister*. Members of the *Lachnospiraceae* family are common human gut microbiota and have been linked to protection from colon cancer (and obesity from the other side), due to their ability to produce butyric acid, which is an important factor for both microbial and host epithelial cell growth [47]. *Dialister* is a Gram-negative member of the Negativicutes class, usually associated with human dental infections [48,49]. We found that *Anaeroplasma* is more abundant in donors A and B, and positively correlates with SYTO9-, PI- cell counts (double negative, which may be considered as not-alive-not-dead). Could those be markers of “good” and “bad” donors? To answer this question, numerous population studies should be performed correlating, e.g., *C. difficile* infection remission with particular donors and particular core microbiota as well as unique bacteria present in their stool.

By comparing flow cytometry with amplicon analysis results, we found a positive correlation between the double negative cell group and *Anaeroplasma* genera. It is possible that, due to their unusual, rich in sterols, cell membrane, members of Mollicutes class, such as *Anaeroplasma*, can be attributed to high numbers of SYTO9-, PI- cells. However, they are definitely not the only ones that built this group, because the relative abundance of Mollicutes was very small in samples from donor C, while SYTO9-, PI- cells per sample percentage in this donor was comparable to others. Nonetheless, there is a possible pattern that high relative abundance of *Anaeroplasma* is more characteristic of donors A and B, being “non-donors” than of donor C, a regular stool donor. It is also possible that double negative cells are bacterial spores.

The selection of an appropriate stool donor is a key step in FMT [50]. Donors are thoroughly screened to ensure they do not harbor any transmittable pathogens or disease. Comparing the gut microbiota profiles of different donors, it is known that microbial diversity is a reliable predictor of FMT success; however, a variety of additional factors, both genetic and environmental, are also known to influence FMT results [51]. As we showed in this work, a regular clinical questionnaire should be coupled with multiple tests results, as only together can they give reliable results, and they will definitely give more data than each method separately.

## 5. Conclusions

Each of the methods described in this study has its pros and cons when applied in the laboratory to characterize the gut microbiota. Classical culturing is relatively cheap and widely available, but it has been shown that culture-based methods can only detect up to 20% of all the bacterial taxa present in a given sample. Methods based on next-generation sequencing are more precise, non-dependent on the cultivation step and can describe every bacterial community with more detail. However, they are also much more expensive, laborious, may introduce PCR biases and do not discriminate between live and dead microorganisms. To tackle this problem, methods such as flow cytometry with fluorochromes (FCM) can be used, as they enable discriminating between live and dead bacteria. Based on this study and the literature searches, it is clear that there is no universal method for the assessment of human gut microbiota, and especially that some methods, such as FCM, may produce only limited results. However, when combined, the methods presented in this study may generate a detailed picture of the fecal microbiota solution for FMT.

In this study, we showed that fecal cultures are characterized by very high variability. By creating a Venn diagram, a “core cultivable microbiota” was identified, and it was composed of only 16 species. The largest number of species, mainly anaerobic, were obtained from donor C, who is a regular donor of stool for FMT. Donor C was characterized by a statistically significantly higher content of species considered beneficial (*Faecalibacterium*, *Bacteroides*, *Barnesiella*, *Blautia*, *Roseburia*, *Butyricimonas*). The general biodiversity of the stool microbiota of donor C was statistically significantly higher than from donors A and B. Microbiota stability over time was also transparent for donor C. In assessing the viability of bacterial cells, three groups of cells were distinguished: alive, dead and unknown. A population of “unknown” cells contained a group of double negative cells (SYTO9-, PI-). The presence of the double negative cell population correlates with the relative amount of *Anaeroplasma*, which appear more frequently in gut microbiota of “non-donors” (donors unsuitable for FMT).

## Supplementary Materials

The following are available online at https://www.mdpi.com/2077-0383/9/7/2036/s1, Table S1: Table depicting raw values obtained from the flow cytometry experiment for all of the investigated samples; Table S2: Table depicting numbers of amplicon sequences remaining after each step of pre-analysis quality filtering.
