# Supplementary material for: Multimodal Approach to Assessment of Fecal Microbiota Donors based on Three Complementary Methods
Source: J Clin Med. 2020 Jun 29;9(7):2036. doi: 10.3390/jcm9072036 (PMC7409046; doi:10.3390/jcm9072036)
Supplement: Supplementary file 1 [file jcm-09-02036-s001.zip › Supplementary matrial description.docx]

**Table S1.** Table depicting raw values obtained from the flow cytometry experiment for all of the investigated samples.

**Table S2.** Table depicting numbers of amplicon sequences remaining after each step of pre-analysis quality-filtering.
